# Computer Vision Tool-Setting System of Numerical Control Machine Tool

**DOI:** 10.3390/s20185302

**Published:** 2020-09-16

**Authors:** Bo Hou, Congpeng Zhang, Shoubo Yang

**Affiliations:** 1 Key laboratory of molecular imaging of Chinese Academy of Sciences, Institute of Automation, Chinese Academy of Sciences, Beijing 100190, China; 2 School of Mechanical and Materials Engineering, North China University of Technology, Beijing 100144, China; soaringroc@ncut.edu.cn; 3 Key Laboratory of Precision Opt-mechatronics Technology, Ministry of Education, Beihang University, Beijing 100191, China; sbyang@buaa.edu.cn

**Keywords:** automatic tool-setting, image processing, intelligent vision machine tool, machine vision, vision measurement

## Abstract

An automatic tool-setting and workpiece online detecting system was proposed to study the key technologies of next-generation intelligent vision computerized numerical control (CNC) machines. A computer vision automatic tool-setting system for a CNC machine was set up on the basis of the vision tool-setting principle. A rapid vision calibration method based on the position feedback from the CNC machine was proposed on the basis of the theory of traditional vision system calibration. The coordinate mapping relationship of the image and the CNC machine, the tool-setting mark point on the workpiece, and the tool tip were calibrated. The vision system performance testing and system calibration experiments were performed. Experimental results indicated that the time consumption was 128 ms in image processing. The precision of tool setting and measuring was less than 1 μm. The workpiece positioning and processing online detection function of the system can completely meet the requirements of visual CNC machine application, and the system has wide application prospects.

## 1. Introduction

**Figure 1:**
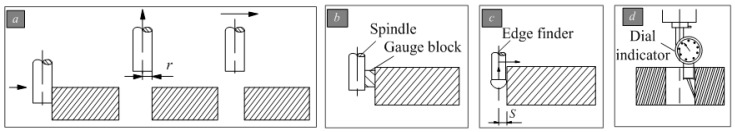
Tool-setting methods: (**a**) manual try turning method, (**b**) standard mandrel method, (**c**) edge finder alignment, (**d**) dial indicator against knife.

Tool setting is a key link in the use of computerized numerical control (CNC) machine tools, and the precision degree of processing parts is decided by the accuracy of the tool setting. Fast and accurate tool setting can greatly reduce machining time and can thus improve the accuracy of product processing. The production efficiency of tool setting is important. The tool setting of the machine tool can be divided into manual and automatic tool setting [1,2]. Examples of manual setting are try turning, feeler gauge, standard spindle, and micrometer methods [3,4], shown in Figure 1.

Trial-cut method (Figure 1a) is a commonly used method in practical applications because of its simple operation. However, the accuracy of the knife is relatively low and cutting marks are produced. The method is suitable for rough machining of knife parts. The production efficiency of the trial-cut method is relatively low. Thus, workers should have a high technical level, otherwise, the quality is difficult to guarantee. As a result, the method is generally used for small batch production. Plug ruler, standard mandrel (Figure 1b), and block gauge tool-setting methods have a simple operation similar to the trial-cut method. They do not produce scratches on the surface of the workpiece. However, the accuracy of the knife and the production efficiency are low. Edge finder, eccentricity bar, and *z*-axis settler are also used for tool setting (Figure 1c). These methods need to use an edge finder and other tools to replace the tool. They have high efficiency and can ensure the accuracy of the knife. Dial (or dial indicator) method (Figure 1d) is mainly used for the tool setting of round workpieces. However, its operation steps are tedious, which result in low efficiency. Nevertheless, its tool-setting accuracy is high. The use of this method on the accuracy of the hole also has high requirements. It is generally used after reaming or boring a hole and cannot be used with a rough hole.

At present, various types of autochecking instruments for tool setting with high accuracy have been widely applied to CNC machine tools [5,6]. An automatic tool counter greatly improves the efficiency of the tool counter to a certain extent. It is easy to use, accurate, automatic, and real time. No technical requirement is needed for the operator [7,8]. However, the knife instrument needs to be equipped with the knife probe separately, and the high-precision knife instrument is costly and difficult to install. Four types of automatic tool-setting instruments are available, namely, plug and pull arms, pull down arms, automatic tool-setting arms, and automatic contact. However, the tool-setting probe must be configured separately, the installation process is troublesome, the tool-setting instrument is costly, and the improvement degree of tool-setting efficiency is limited [9,10].

In summary, the traditional manual knife method is relatively simple to operate. However, the method has poor security, relatively long knife time, and relatively large random errors due to human factors. These shortcomings are difficult to adapt to the pace of CNC machining and greatly reduce the efficiency of the machine tool. Moreover, a manual knife has low precision, long knife time, and very low efficiency. The price of an automatic knife instrument is also costly, and the installation is troublesome. Thus, the method is unsuitable for large-scale widespread application. Therefore, a high-precision automatic tool-setting method for CNC machine tools should be developed.

At present, the technologies of computer machine vision have become a key part in next-generation intelligent vision CNC machines with the advantages of high precision, high efficiency, noncontact, and intelligence [11,12,13,14,15]. These technologies have attracted considerable attention from local and international scholars. J. et al. [16] presented a new measurement system that quantifies broaching tool wear on the basis of the overall wear area. The proposed method uses automated image cropping and digital imaging processing tools to determine the affected area without requiring any manual intervention. ZHANG et al. [17] investigated the algorithms of tool setting for PCB (Printed Circuit Board) milling cutter sharpening based on machine vision. Canny edge detection and Harris corner detection algorithms were used to find the image feature points under different types of milling cutter. The angle of tool setting was obtained according to the geometric relationship of image feature points. The results showed that the maximum error of angle of tool setting while PCB milling cutters are in the same position can be achieved with a repetition accuracy of no more than 0.370°. QIN et al. [18] proposed a visual detection method for tool wear state in metal cutting process. This method results in fast and accurate tool state detection. Fernández-Robles et al. [19] presented a reliable machine vision system to automatically detect inserts and determine if they were broken. The aforementioned studies show that tool identification and workpiece positioning based on computer vision technology have been well-applied in intelligent control and closed-loop acknowledgement of CNC machine tools. They also provide valuable references for computer vision automatic tool setting.

Machine vision technology simulates human vision through a computer, presents objective things in the form of images, recognizes and extracts information from the images, processes and analyzes them, and finally uses them for detection, measurement and control. A typical machine vision system consists of four parts: a computer, a camera, a frame grabber, and a light source. Online detection of workpiece and tool in the CNC machining process is combined with visual feedback to realize automatic tool setting. The holographic control of the production process can also be realized, Kim et al. [20] mentions that the video information is fed back to the control room to provide human eyes with real-time monitoring of CNC status. Chang et al. [21] focuses on the three-dimensional reconstruction method of the workpiece position. Jang et al. [22] mentions a workpiece positioning method, and the positioning application of vision technology in parts processing is proposed. The traditional technique of tool setting can only determine the coordinate values of the cutter. However, the vision system not only can measure the tool coordinates but also can realize the information positioning and online detection of the machining process [23]. Therefore, a fast calibration method and system for a vision system based on position feedback of the machine tool is proposed in the present study on the basis of vision and image technology. The principles and technology of automatic tool setting of CNC machine tools based on computer vision are also explored. The proposed method can finish the automatic tool setting with high precision and efficiency, improve the quality and efficiency of NC (Numerical Control) processing by establishing the coordinate transformation relationship among “visual image”, “machine tool (tip)”, and “workpiece”. The method also has its own theoretical value and practical importance.

Aiming at the urgent industry application demands of automatic high-precision CNC machine tool setting, in addition, the harsh working conditions of CNC machining pose a major challenge to the optical inspection system. This paper introduces a fast calibration method and system for a vision system based on position feedback of the machine tool. The contributions of this study are listed as follows:

(1) Tool setting is an essential primary operation in CNC machining due to the low precision and efficiency of traditional manual tool setting methods and the high price and low efficiency of automatic tool-setting instruments in the industry. This paper presents a method of automatic tool setting for CNC machine tools. This method can achieve high precision and fast automatic tool setting in contactless conditions.
(2) A rapid vision calibration method based on the position feedback of the CNC machine tool is proposed to address the problem of the limited activity space of a CNC machine. A calibration board and other targets are not required, and the calibration of the vision system can be conducted rapidly with the coordinates of the CNC machine tool and the system motion model.
(3) A computer vision automatic tool-setting system for a CNC machine is developed. The system has a simple and efficient tool-setting process and achieves an accuracy of <1 μm. The time required for the image acquisition and processing of the vision system is 128 ms.

The remainder of the paper is organized as follows. Section 2 mainly describes the principal theories, including the visual automatic tool-setting method based on the traditional tool-setting process, the composition of the visual system, and the basic process of tool setting. In Section 3, the calibration of the vision system and the rapid calculation method of the coordinates of the tool-setting mark point are introduced. The experimental results with real data are provided in Section 4. Conclusions are drawn in Section 5.

## 2. The Principle of Vision Automatic Knife Alignment

### 2.1. Subsection Principles of Tool Setting in CNC Machining

When the workpiece is clamped onto the machine tool, the correct position of the workpiece on the machine tool must be determined. The process of location is achieved by tool setting, that is, accurately locating the “cutter position point” to the position of the “tool-setting point” [24]. The “cutter position point” is the datum point to determine the position of the cutter. The lathe tool cutter position point is the tip, the drill cutter position point is the drill point, the flat-end milling cutter position point is the center of end surface, and the ball-end milling cutter position point is a sphere. “Tool-setting point” refers to the starting point of the cutter relative to the workpiece machining movement. In general, the tool-setting point is selected as the design basis of the workpiece. For a square workpiece, the intersection of the right end face of the workpiece and the center line is usually selected as the tool-setting point in the CNC lathe. The intersection point of the two vertical sides of the workpiece is taken as the tool-setting point in the CNC milling machine. For a round workpiece, the center is often selected as the tool point.

**Figure 2:**
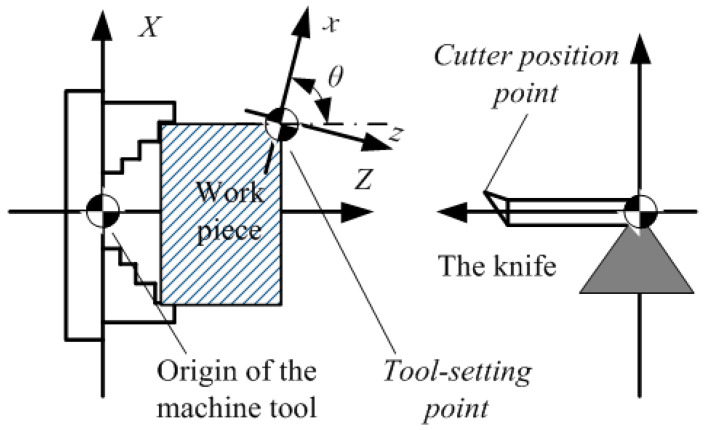
Principles of tool setting in CNC machining.

As shown in Figure 2, the two key coordinate systems for CNC machining and tool setting are the machine tool coordinate system (*O-XYZ*) and workpiece coordinate system (programming coordinate system, *o-xyz*). The machine tool coordinate system is defined before the machine tool leaves the factory, and this system can be regarded as the world coordinate system. The position of the cutter-starting point is the reference point coordinate system of the machine tool, that is, the coordinate value of the starting point displayed on the control screen of the machine tool must be determined to accurately locate the cutter position point to the cutter-starting point. Therefore, the essence of tool setting is to measure the distance bias between “tool-setting point” and “cutter position point” according to the distance bias to set program origin (tool-setting point) coordinates in the machine tool coordinate system. The coordinates of the cutter position point are (*X*, *Y*, *Z*), and the coordinates of the tool-setting point are (*x*, *y*, *z*). The tool-setting model of CNC machine can be expressed as Formula (1).

(1) $$\{\begin{array}{l} x=X+\Delta X\\ y=Y+\Delta Y\\ z=Z+\Delta Z \end{array}$$

where ΔX, ΔY and ΔZ are the offset of the tool-setting point relative to the origin of the machine tool coordinate system that need to be solved.

The theories of computer vision automatic tool setting include a visual acquisition tool, workpiece image information, image processing, and feature point identification. They are combined with the known coordinate system of the machine tool. The transformation relationship of image–machine–workpiece is established through the space coordinate mapping, and the CNC machine tool automatic tool-setting program is realized according to the image information to determine the tool-setting mark point coordinates.

### 2.2. Computer Vision Automatic Tool-System

**Figure 3:**
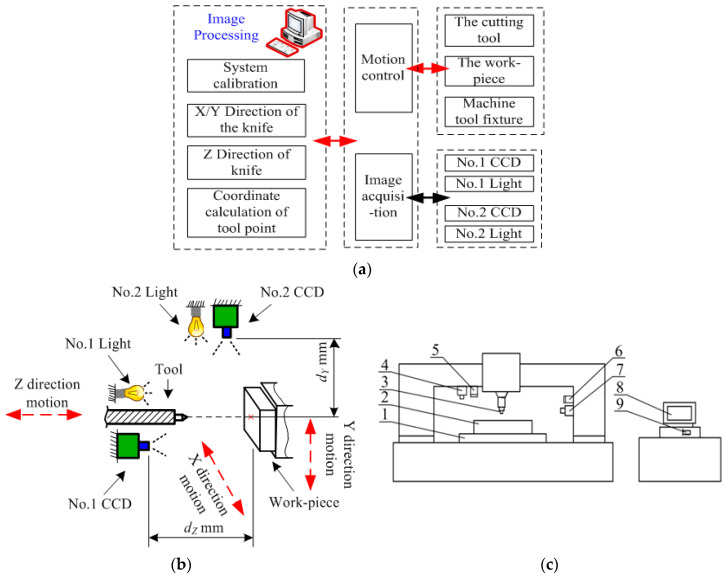
(**a**) CNC machine tool visual tool system composition, (**b**) CCDs installation structure diagram, (**c**) 1—CNC machine tool table, 2—workpiece, 3—tool, 4—No. 1 CCD, 5—No. 1 light, 6—No. 2 light, 7—No. 2 CCD, 8—computer, 9—image capture card.

With respect to the *X*, *Y*, and *Z* coordinates of tool setting of a CNC machine tool, at least two sets of visual acquisition systems should be configured to obtain image information of tool-setting mark points from three degrees of freedom. Therefore, a visual tool-setting system for CNC machine tools, as shown in Figure 3, is designed.

In Figure 3a, in the form of a block diagram, the vision automatic tool-setting system is reviewed, the left side of the figure for part of the image processing algorithm, the right to exercise the module of the image acquisition module and the hardware part, the movement module including the cutting tool, workpiece and machine tool fixture, the image acquisition module includes No. 1 CCD (Charge Coupled Device), No. 1 Light, No. 2 CCD and No. 2 Light. Figure 3b shows the installation form of No. 1 CCD and No. 2 CCD on the machine tool with the three-dimensional schematic, where, *d_Z_* represents the working distance of No. 1 CCD image acquisition, and *d_Y_* represents the working distance of No. 2 CCD image acquisition. The hardware and installation structure diagram of the vision automatic tool-setting system are shown in Figure 3c.

The system is equipped with two high-resolution industrial cameras with low-distortion lenses. The micro CNC machine system is installed in horizontal and vertical directions, with the horizontal direction being the No. 1 CCD and the No. 1 light, the vertical direction being the No. 2 CCD and the No. 2 light. The No. 1 CCD obtains the image information of cutter-workpiece in *X* and *Y* directions, and the No. 2 CCD obtains the image information of cutter-workpiece in *Z* direction. After the cameras are fixed, the relative position of the CNC machine tool remains unchanged.

### 2.3. Flows of Computer Vision Tool-Setting System

On the basis of the computer vision tool-setting system shown in Figure 1, the basic flow of visual tool-setting is described as follows:

(1) Computer vision system calibration: with the calibration of the computer visual system, distortion correction of the visual system is accomplished on the one hand, and the coordinate space conversion relations between No. 1 CCD and the CNC system are determined on the other hand. This process can only be calibrated after the installation of the visual system, and calibration operation is unnecessary if the relative position of the visual system and the CNC machine tool does not change.
(2) The tool-setting process in *X* and *Y* directions based on the selected workpiece tool-setting mark point: the clear image, which shows the tool point, is collected by the No. 1 CCD and is processed to obtain the image coordinates of the tool-setting mark point in *X* and *Y* directions.
(3) The tool-setting process in *Z* direction: the tool tip is selected as the tool-setting mark point in *Z* direction. The clear image, which shows the tip and workpiece surface, is collected by the No. 2 CCD for image processing to obtain the information from the tip to the tool-setting plane in *Z* direction.
(4) The 3D coordinate value, which is obtained by solving the tool-setting mark point relative to the tool tip in the coordinate system of the machine tool, is combined with the image coordinates of the tool-setting mark point and the system calibration result. The automatic tool setting is completed in the machining process.

After establishing the computer visual numerical control system and the first system calibration, the automatic tool setting and workpiece positioning during the machining process are completed by steps (2) to (4) after each workpiece replacement.

## 3. Calibration of Visual Tool-Setting System

In this study, a visual system calibration method based on machine tool coordinate information feedback is proposed on the basis of the theory of image measuring and traditional camera calibration methods [25,26,27,28]. The parameters, such as cutter displacement coordinate and mark point coordinate for calibration, are obtained from position feedback of the numerical control system. A special calibrated displacement measurement system need not be installed. Thus, the calibration process is simple and efficient and is helpful in the engineering application of visual tool setting.

### 3.1. Image-Machine Coordinate Mapping

**Figure 4:**
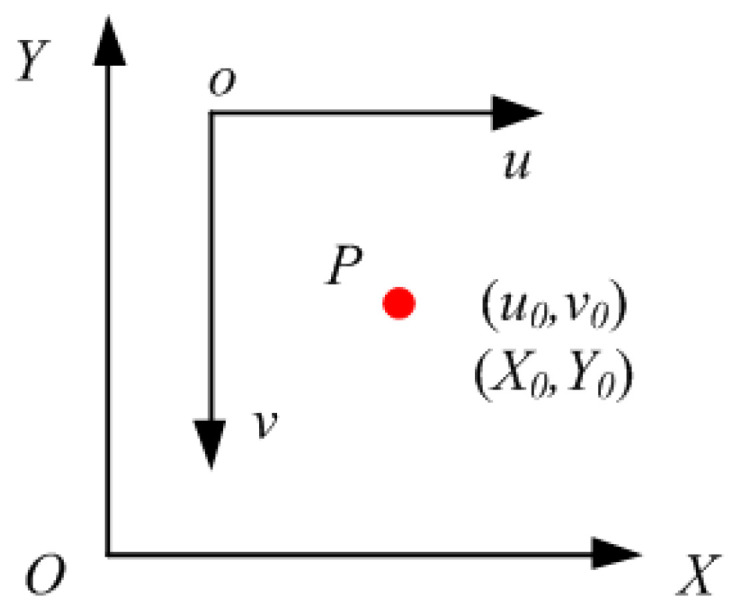
Machine tool coordinate system (*XOY*) and image coordinate system (*uov*). A point on the workpiece is chosen as an image processing mark point. The image coordinates of subpixel corner points (*u*_1_,*v*_1_) are obtained from No. 1 CCD, and mark points are processed.

The image–machine coordinate mapping relationship is shown in Figure 4 for establishing the transformation relation model from image pixel coordinate system to machine tool coordinate system. The *u*-axis of the image coordinate system is positively correlated with the *X*-axis of the physical coordinate system, while the *v*-axis of the image coordinate system is negatively correlated with the *Y*-axis of the physical coordinate system. The coordinates of the set point P in the image coordinate system and the machine coordinate system are (*u*_0_,*v*_0_) and (*X*_0_,*Y*_0_), respectively. Figure 4 contributes to the subsequent transformation model of the image coordinate system to the physical coordinate system. Accordingly, the visual system can calculate the machine coordinate information from the image coordinate. Physical sizes of pixels *k_x_, k_y_*, and *k_z_* are calculated by means of tool-setting point movement, that is, the CNC machine tool controls the cutter movement. A mapping relationship is established by the coordinate value, which displays the machine tool and the image pixel coordinate value of the current cutter.

**Figure 5:**
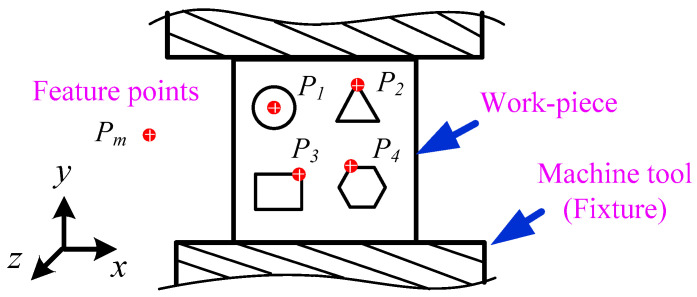
Schematic of an image captured by No. 1 CCD.

Figure 5 shows the schematic of the image acquired by camera 1. The acquisition field is mainly the workpiece mounted on the fixture. Feature points Pm on the workpiece can be set by ourselves and are determined by the image processing algorithm. For circular parts, feature points (*P*_1_) can be selected as the center of the circle, and the center extraction algorithm can be used for image processing. For polygonal parts, corner points can be selected as feature points (*P*_2_, *P*_3_ and *P*_4_) and corner point algorithm is adopted for image processing et al.

The micro distance movement of the machine tool is controlled along the horizontal direction, images are collected, and the image coordinates (*u*_2_,*v*_2_) of mark points are extracted again. Multiple cycles are conducted to reduce the lens distortion error and random error of the calibration process. The values of *k_x_* and *k_y_* are calculated by Formula (2):

(2) $$k_{x}=\overset{n}{\underset{i=0}{\sum }}\frac{X_{i+1}-X_{i}}{\sqrt{{\left(u_{i+1}-u_{i}\right)}^{2}-{\left(v_{i+1}-v_{i}\right)}^{2}}}/n\;k_{y}=\overset{n}{\underset{j=0}{\sum }}\frac{Y_{j+1}-Y_{j}}{\sqrt{{\left(u_{j+1}-u_{j}\right)}^{2}-{\left(v_{j+1}-v_{j}\right)}^{2}}}/n$$

where *n* is test times, *X_i, j_* and *Y_i, j_* are machine tool coordinates, *u_i, j_* and *v_i, j_* are image coordinates.

The relationship transformation of image and machine tool coordinates is established on the basis of *k_x_* and *k_y_*. The machine tool and image coordinates of point P are (*X*_0_, *Y*_0_) and (*u*_0_, *v*_0_), respectively. The image of the calibration workpiece is collected by the No. 1 CCD, and the image coordinates of its mark points are extracted. The calibration of the mapping relation between image and machine tool coordinates is completed and combined with the current coordinate value of the machine tool.

(3) $$\begin{array}{l} X_{1}=u_{1}\times k_{x}-[(u_{0}\times k_{x}+\Delta X)+X_{0}]\\ Y_{1}=v_{1}\times k_{y}-[(v_{0}\times k_{y}-\Delta Y)-Y_{0}] \end{array}$$

The motion variable Δ is introduced to express the image–machine tool coordinate mapping relationship in *X* and *Y* direction, as shown in Formula (3), for increasing the applicability of coordinate transformation. In the formula, Δ*X* and Δ*Y* are the displacements of the machine tool in *X* and *Y* directions, respectively, during the calibration.

**Figure 6:**
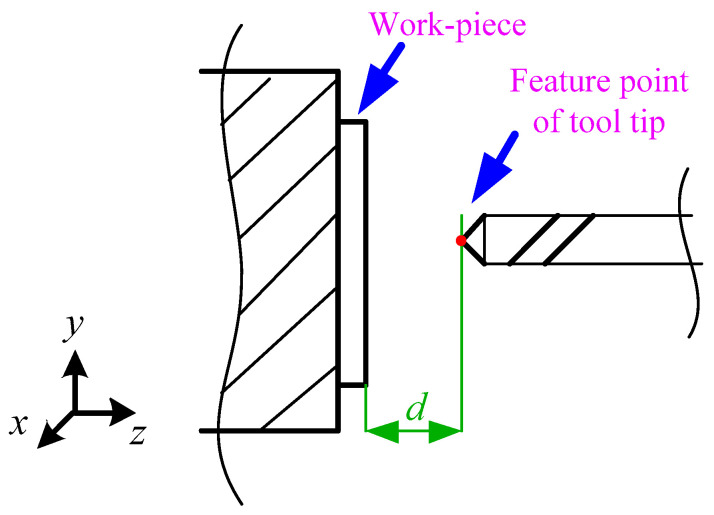
Schematic of an image captured by No. 2 CCD.

The control machine tool conducts micro distance movement along the *Z* direction. Images containing the tool tip and the workpiece coordinate are collected by the No. 2 CCD. Figure 6 shows the schematic of the image acquired by No. 2 CCD. The acquisition field includes the tool and the tool plane of the workpiece mounted on the fixture. The distance between the characteristic point of the knife tip and the plane of the opposite knife can be expressed as d. On the basis of the image coordinates (*u_z_*, *v_z_*) of the feature points of the tool tip, the pixel size *k_z_* is calculated by Formula (4).

(4) $$k_{z}=\sum_{i=1}^{n}\frac{Z_{i+1}-Z_{i}}{\sqrt{{\left(u_{i+1}-u_{i}\right)}^{2}-{\left(v_{i+1}-v_{i}\right)}^{2}}}/n$$

where *Z_i_* is the machine tool coordinate, and *u_i_*, and *v_i_* are image coordinates.

The distance *d_m_* from the tip point to the surface of the tool is measured in *Z* direction, and the distance formula from the point to the line is solved in the image coordinate system. As shown in Formula (5), the linear equation of the projection of the workpiece surface image is fitted when location of the workpiece is fixed. *A*, *B*, and *C* in the formula are equation constants.

(5) $$d_{m}=\frac{\left|Au_{z}+Bv_{z}+C\right|}{\sqrt{A^{2}+B^{2}}}$$

### 3.2. Tool-Setting Mask Point-Tool Tip Point Coordinate

The positional relationship between the tool tip and the workpiece is an important basis for the conversion of the tool-setting mark point image coordinates to the machine coordinates. The implementation process is described as follows:

(1) The numerical control machine tool controls the in-feed, and a circular hole is cut perpendicular to the surface of the workpiece, and the circular hole has a circular feature on the image plane. The No. 1 CCD captures the workpiece image and records the *X_0_* and *Y_0_* values in the machine coordinate system at this time.
(2) The image information of the circular hole feature is extracted, and the image coordinate (*u_0_, v_0_*) of the center of the circular hole, that is, the projection of the tool tip on the surface of the workpiece, is fitted. The offset *u_p_* and *v_p_* between the projection point and the tool-setting mark point coordinate (*u_1_, v_1_*) in the *X* and *Y* direction is shown in Formula (6).
  (6) $$u_{p}=u_{1}-u_{0},v_{p}=-v_{1}+v_{0}$$
(3) The offset of the center of the tool setting in the image–machine coordinate mapping relationship in the *X* and *Y* direction is introduced, and the transformation of pixel coordinates of tool-setting mark point on the workpiece in *X* and *Y* direction to the machine tool is completed. The mapping of the fully calibrated image coordinates to machine tool coordinates is shown in Formula (7).
  (7) $$\begin{array}{l} X_{1}=X_{0}+u_{p}\times k_{x}=u_{1}\times k_{x}-[(u_{0}\times k_{x}+\Delta X)+X_{0}]\\ Y_{1}=Y_{0}+v_{p}\times k_{y}=v_{1}\times k_{y}-[(v_{0}\times k_{y}-\Delta Y)-Y_{0}]\\ Z_{1}=Z_{0}-d_{m}\times k_{z}=Z_{0}-k_{z}\times \frac{\left|A_{0}u_{z}+B_{0}v_{z}+C_{0}\right|}{\sqrt{A_{0}{}^{2}+B_{0}{}^{2}}} \end{array}$$

### 3.3. Acquisition of Coordinates in the X and Y Directions

**Figure 7:**
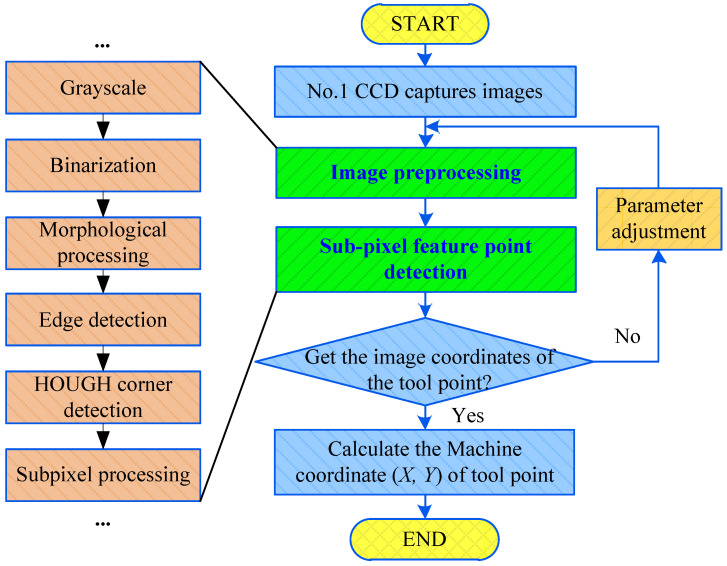
Flowchart of extracting *XY* coordinate from the tool-setting point.

The extraction process of *X* and *Y* coordinates of the tool-setting mark point is shown in Figure 7 No. 1 CCD collects the image containing the knife point for image preprocessing (e.g., grayscale and binarization). The subpixel coordinates of the feature points [29] on the image are extracted. When the image coordinates of the feature points are obtained, the machine tool coordinates of the feature points can be calculated using the coordinate transformation model described in this paper (Equation (7)).

### 3.4. Acquisition of Coordinates in the Z Direction

The No. 2 CCD collects images which show the tool tip and the tool-setting plane and adopts a template matching algorithm [30,31] to identify the tool tip. The corresponding tool tip templates are set for different tools, and the matching results preliminarily locate the tool position. Subpixel edge information of the region is extracted according to the coarse positioning results. The projection of the fitting workpiece onto the tool-setting plane is a straight line, and the pixel distance from the tool tip to the fitting line is solved by the ranging principle. The shortest pixel point from the tool-setting plane is determined to be the tool tip for completing the accurate positioning of the tool tip.

**Figure 8:**
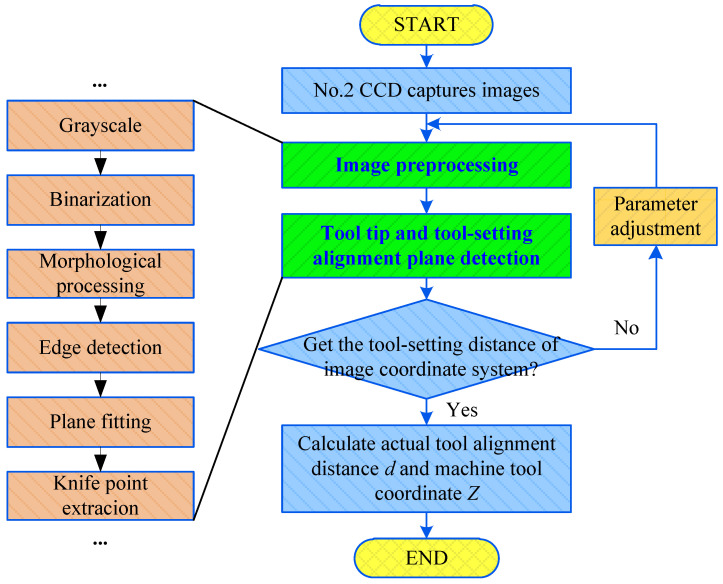
Flowchart of extracting *Z* coordinate from the tool-setting point.

The algorithm for acquiring the *Z* coordinate of machine tool is shown in Figure 8. First, the No. 2 CCD collects the image information for the tool tip and tool-setting plane. Then, the image distance from the tool tip to the projection line of the tool-setting plane is calculated by Formula (5). Finally, the *z* coordinate value of tool-setting mark point in the machine tool coordinate system is calculated by Formula (7).

## 4. Experiment and Result Analysis of Automatic Tool Setting

### 4.1. Experimental System

**Figure 9:**
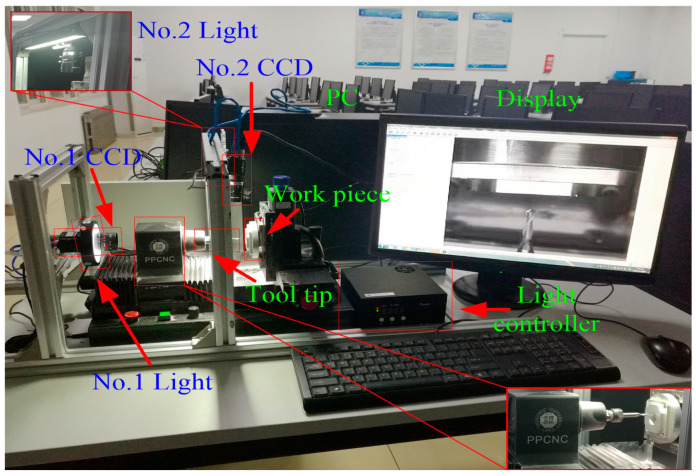
System for automatic tool-setting experiment.

In this study, a computer vision automatic tool-setting system for a CNC machine was built on the basis of a micro CNC milling machine PPCNC (Personal Portable CNC Machine). The experiment platform is shown in Figure 9. The installation method of Nos. 1 and 2 CCD was consistent with that described in Figure 3. No. 1 CCD and No. 1 light were mounted horizontally along the cutting tool direction, No. 2 CCD and No. 2 light were installed vertically along the vertical cutting tool direction. The configuration of each hardware module is shown in Table 1. The resolution of the micro CNC milling machine was 1 μm, and the precision of the vision system was expected to be 1 μm. We used the industrial camera GS3-U3-91S6M-C (Manufacturer: FLIR, Richmond, Canada) with a resolution of 3376 × 2704 and the supporting industrial lens V5028-MPY (Manufacturer: Computar, Tokyo, Japan). With a field of view of 4 mm, the physical accuracy was calculated to be approximately 1.18 μm. Then, four times the subpixel interpolation detection was used, and the theoretical accuracy should reach 0.295 µm. In accordance with Figure 3b, we installed the No. 1 CCD at a distance *d_Z_* of 150 mm and the No. 2 CCD at a distance *d_Y_* of 100 mm due to the constraints of the working conditions. In addition, No. 1 light source was ring white light and No. 2 light source was set by the array light source in order to ensure the field of view of the No. 2 CCD.

### 4.2. Experiment of Calibration Relationship of Vision System

**Figure 10:**
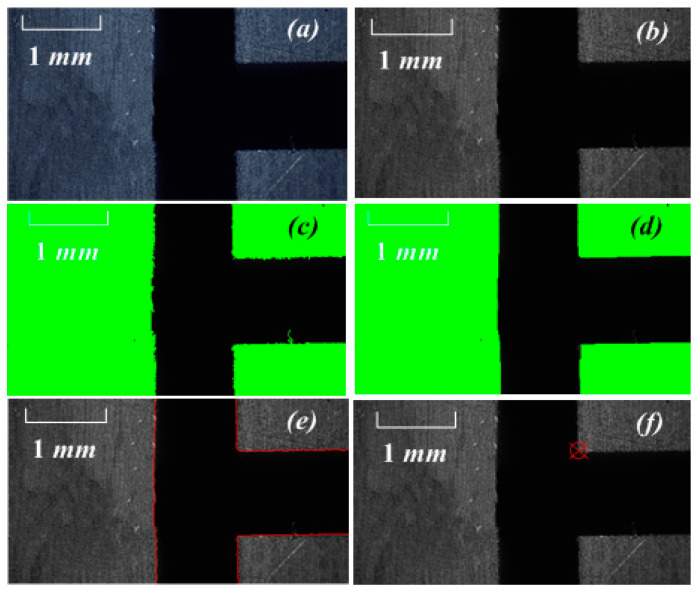
Experiment part and cutting point: (**a**) original image, (**b**) gray image, (**c**) threshold of binary image, (**d**) erosion and dilation operations in binary mathematical morphology, (**e**) edge detection, (**f**) image coordinate acquisition of feature points by edge fitting.

The calibration method based on position feedback of CNC machine tool was used to determine the mapping relationship of the experimental vision system. The CNC milling machine was operated to ensure that the workpiece was in the field of vision of the No. 1 CCD and that one mark point of the T-groove was the tool-setting mark point, as shown in Figure 10. The image processing process of extracting feature points of round holes and corner points from the image obtained by No. 1 CCD. The process included but was not limited to grayscale, binarization, morphological processing, edge detection, Hough corner detection, and subpixel processing. This procedure was done using the C# + EmguCV image processing function library. The main content of this article was the extraction of visual automatic knife method. Therefore, the implementation details of image processing are only briefly described.

The image coordinates (*u_i_*, *v_j_*) of the tool-setting mark point and the precision of the subpixel were extracted. The coordinates (*X_i_*, *Y_j_*) of the machine tool were also recorded. The workpiece was moved along the x direction to extract (*u_i+_*_1_,*v_j+_*_1_), (*X_i+_*_1_,*Y_j+_*_1_). The experimental data are shown in Table 2.

The workpiece was moved along the y direction. The image coordinates of characteristic mark point were extracted, and the corresponding coordinates of machine tool were recorded. The experimental data are shown in Table 3.

After the experimental data were processed, we used Formula (1) to obtain the pixel size values of No. 1 CCD:

*k_x_* = 0.000758 mm/pixel

*k_y_* = 0.000757 mm/pixel

**Figure 11:**
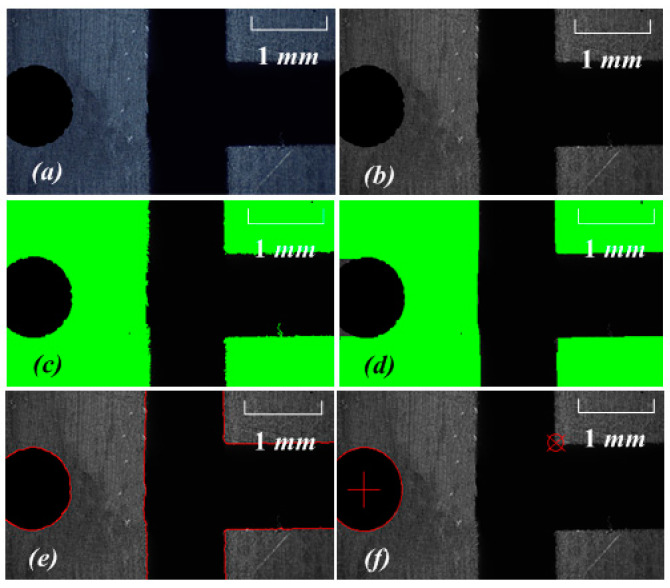
Round hole cut by the cutting tool for experiment: (**a**) original image, (**b**) gray image, (**c**) threshold of binary image, (**d**) erosion and dilation operations in binary mathematical morphology, (**e**) edge detection, (**f**) image coordinate acquisition of feature points by edge fitting.

The workpiece was controlled to enter the area with a clear tool-setting mark point. The machining tool was used to make a round hole from the calibrated workpiece. The coordinates of the machine tool at this time were recorded as (24.000 mm, 8.000 mm). The image collected by No. 1 CCD, and the image processing process is shown in Figure 11. The image processing of a tool trial-cut round hole was added to provide the tool with an initial positioning coordinate point in the *X/Y* direction of the workpiece. Thus, the original data source for *X_0_/Y_0_* in Formula 7 was provided. The tool-setting flow and image processing in the *X* and *Y* direction are described by Figure 10 and Figure 11.

In the image coordinate system, the subpixel coordinates of the mark point and the center of the round hole were extracted. The coordinates of the tool-setting mark point were (915.859 pixel, 1684.798 pixel), and those of the center of the round hole were (337.214 pixel, 1604.373 pixel).

**Figure 12:**
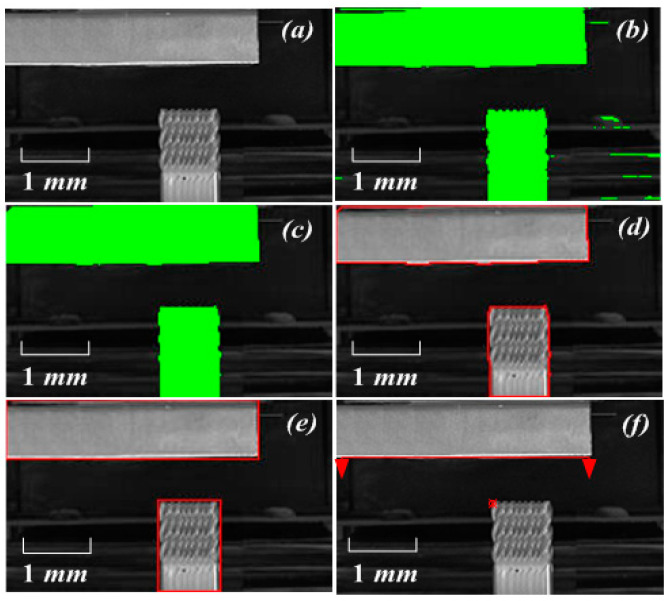
Obtaining the tip by image processing: (**a**) original image (**b**) threshold of binary image, (**c**) erosion and dilation operations in binary mathematical morphology, (**d**) edge detection, (**e**) edge fitting, (**f**) alignment plane and alignment point.

The tool moved along the *Z* direction. The No. 2 CCD collected the images with the tool tip point, extracted the image coordinates of the tool tip point, and recorded the corresponding machine coordinates. The image processing is shown in Figure 12, the process included but was not limited to grayscale, binarization, morphological processing, edge detection, plane fitting, and knife point extraction. The experimental data are presented in Table 4. The pixel equivalent value *k_z_* was calculated using Equation (2):

*k_z_* = 0.000782 mm/pixel

**Figure 13:**
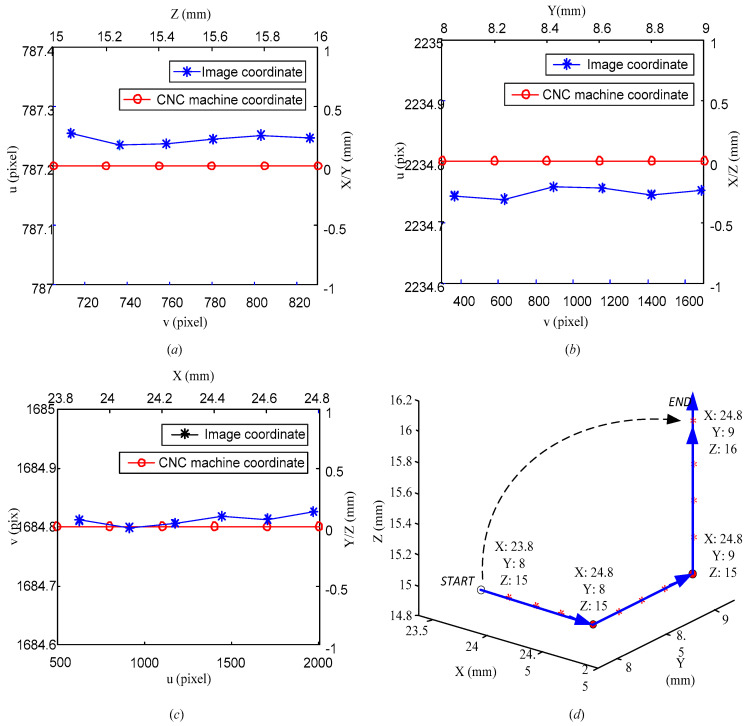
(**a**) Data of Table 2, (**b**) data of Table 3, (**c**) data of Table 4, (**d**) trajectory of CNC machine tool.

Figure 13 shows the statistical chart corresponding to the coordinates of the machine tool and the extracted image for Table 2, Table 3 and Table 4. Figure 13d depicts the three-dimensional motion trajectory of the machine tool during the entire tool-setting process. The order of the movement direction of the machine tool may not be limited but should relate to the corresponding image algorithm.

### 4.3. Tool-Setting Mask Point Coordinate Extraction

The CNC machine coordinates of the workpiece tool-setting mark point can be calculated using the experimental data. The specific process is described as follows:

(1) The No. 1 CCD captures the image and extracts the subpixel precision coordinates of the tool-setting mark point. The coordinates are (871.356 pixel, 447.223 pixel). The values of *X* and *Y* of the CNC machine coordinate of the tool-setting mark point at this position can be calculated as (26.713 mm, 8.684 mm) by Formula (7).
(2) The No. 2 CCD captures the image and identifies the tool tip point by tool template matching, as shown in Figure 12. The image subpixel precision coordinates of the tool tip point are extracted as (799.585 pixel, 909.206 pixel). At the same time, the tool-setting plane straight line of the workpiece is fitted. The linear equation obtained in the image coordinate system of the No. 2 CCD is 5.267x + 424.167y − 295653.033 = 0. The image distance d from the tool tip point to the tool-setting plane is calculated by Formula (5), and the *Z* coordinate of the CNC machine corresponding to the tool-setting mark point at this position is calculated as 24.733 mm by Formula (7).

### 4.4. Verification of the Accuracy of the Vision System

The accuracy of the vision tool-setting system was evaluated from two aspects: repeatability and precision of detection. Firstly, the repeatability of the vision system was verified through experiments. The experimental steps are detailed as follows:

(1) The CNC machine is controlled to slightly move the workpiece in the *XOY* plane. The CNC machine coordinates after the movement are different from the CNC machine coordinate when the hole is cut. The current coordinates (*X’, Y’, Z’*) of the CNC machine are recorded.
(2) Subpixel feature extraction is performed on the image of the No. 1 CCD, and the image coordinates (*u’, v’*) of the center of the trial-cut feature hole in the CNC machine coordinates are recorded. The image coordinates (*u_0_, v_0_*) of the center of the hole are obtained during the initial trial cutting of the tool as (337.214 pixel, 1604.374 pixel). Thus, the image coordinate difference between the center of the current hole and the center of the hole in the initial trial state can be calculated.
  (8) $$\begin{array}{l} x_{v}=\left|\Delta X-k_{x}*\Delta u\right|=\left|\left(X’-X_{0}\right)-k_{x}*\left(u’-u_{0}\right)\right|\\ y_{v}=\left|\Delta Y-k_{y}*\Delta v\right|=\left|\left(Y’-Y_{0}\right)-k_{y}*\left(v’-v_{0}\right)\right|\\ z_{v}=\left|\Delta Z-k_{z}*\Delta d\right|=\left|\left(Z’-Z_{0}\right)-k_{z}*\left(d’-d_{0}\right)\right| \end{array}$$
(3) The CNC machine is controlled to move slightly in the *Z* direction. The *Z* coordinates of the CNC machine before and after the movement are recorded. The tool tip point and workpiece tool plane information of the No. 2 CCD image in the two coordinates are extracted. The image distance d of the tool-setting movement is calculated by Formula (5), and the direction error is calculated by Formula (8).

**Figure 14:**
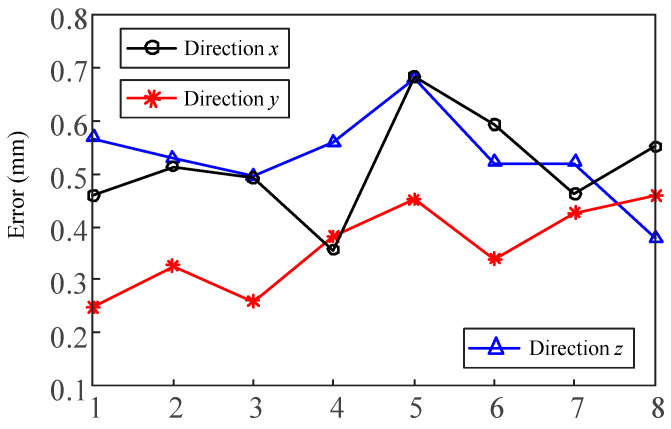
Statistical graph of direction error.

The verification results measured by vision system are shown in Table 5 and Table 6, and the end of the table presents their statistics results. Figure 14 shows the statistics chart of direction error. A total of eight series of vision automatic tool-setting tests were completed. In general, the tool-setting error of the system in three directions was relatively stable, and the statistical values of mean, std and RMS (Root Mean Square) performed well.

Secondly, the author used the laser measuring sensor ConoPint-3 (Manufacturer: OPTIMET, Jerusalem, Israel) with accuracy of micrometers for comparison and verification of the accuracy of the tool-setting results in this experiment. ConoPint-3 uses a unique conoscopic holographic technology for distance measurement. Compared with the standard triangulation method, the sensor has the advantages of collinearity and low electronic noise dependence, and its measurement accuracy can reach 1 μm. Table 7 presents a list of the sensor parameters.

**Figure 15:**
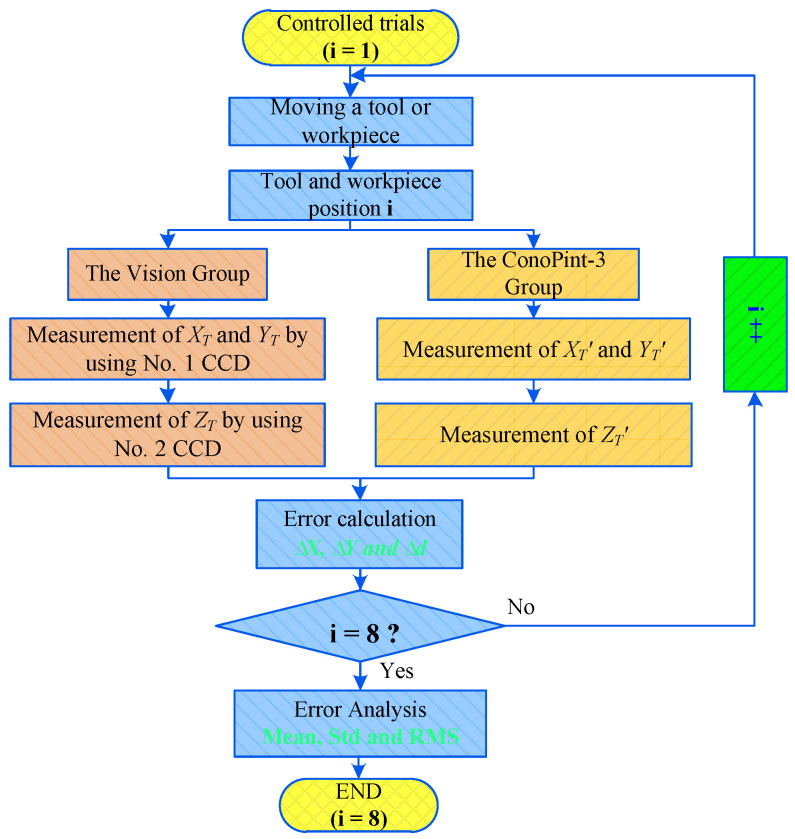
Controlled trial workflow.

**Figure 16:**
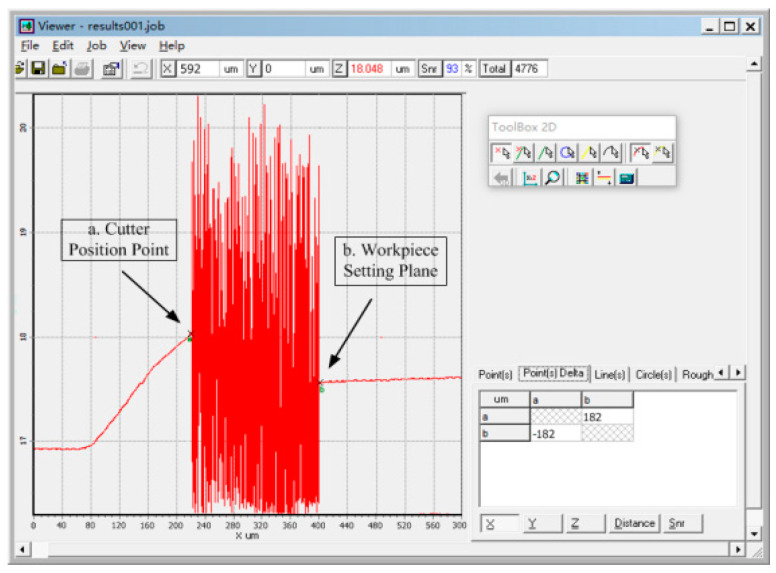
Screenshot of the software running when ConoPint-3 is measuring in the Z direction.

The accuracy of the tool setting could be clearly reflected by the measurement accuracy. We used the sensor to measure the distance from the tool to the surface of the workpiece, including the measured values in the three directions of *X*, *Y*, and *Z*. ConoPint-3 adopts the laser measurement principle, and its installation method is the same as that of the camera in this study. It was installed along the horizontal and vertical directions to measure the distance between the tool and the feature points of the workpiece in the *X*, *Y*, and *Z* directions. The controlled trial process is shown in Figure 15. In particular, the tool or workpiece was moved to the sensor acquisition range in position 1 (i = 1). The vision group No. 1 CCD collected and processed the tool tip to the feature point of the workpiece in the *X* and *Y* directions, and the distance was expressed as *X_T_*, *Y_T_*. The No. 2 CCD collected and processed the distance *d_T_* between the tool tip and the tool-setting plane in the Z direction. The ConoPint-3 sensor also synchronously measured the values in the *X*, *Y*, and *Z* directions, which were expressed as *X_T_ ‘, Y_T_’*, and *d_T_’*, respectively, and calculated the measurement errors ∆*X*, ∆*Y*, and ∆*d*. It completed a set of control measurement experiments. Figure 16 is a screenshot of ConoPint-3 software running in the *Z* direction, where the mark point a represents the tool tip point, point b represents the workpiece tool-setting plane, and the horizontal distance from *a* to *b* (corresponding to the *X* direction of the sensor) represents the distance from the tool to the workpiece The distance was 182 μm. The irregular pulse stripes in the middle area were air gaps. The tool or workpiece was moved to change the spatial position (i + 1) of the two. The comparison test was repeated eight times, and the error mean, Std, and RMS values were calculated and analyzed.

Table 8 shows the experimental data obtained using ConoPint-3 and via vision-assisted tool-setting measurement to verify the accuracy of the measurement results of the vision system. The results showed that the test results of the two measurement methods were basically the same, the average error was less than 0.15 μm, and the Std and RMS performance were good. Compared with ConoPint-3 assisted tool setting, the vision system had superior performance, which was mainly reflected in the higher submicron measurement accuracy and smarter feature point extraction method. The measurement process, such as manual feature point selection, of Conopint-3 was also more time consuming.

After completing the experiment of repeatability and precision of detection, the results showed that the visual tool-setting system based on the CNC micro milling machine realized automatic tool setting based on the visual measurement. The system achieved an accuracy of less than 1 μm. The time required for the image acquisition and processing of the vision system was 128 ms. The accuracy and speed of the system meet the requirements of modern manufacturing.

## 5. Conclusions and Outlook

### 5.1. Conclusions

This study analyzes the advantages and disadvantages of the commonly used tool-setting methods in numerical control machine tools. An automatic tool-setting method in a numerical control machine tool based on computer vision is proposed. An automatic tool-setting system with high efficiency and precision was developed. The system calibration and computer vision tool-setting measurement experiments were completed. The experimental results showed that the vision-based tool-setting scheme can realize automatic tool setting of the micro milling machine with short time consumption and high positioning accuracy. The efficiency of the scheme is nearly 100 times higher than the traditional tool-setting scheme. For micro precision parts, tool-setting accuracy of less than 1 μm can be achieved through specific calibration parts. This accuracy meets the application requirements of numerical control machine tools. The system can be used for online positioning detection and noncontact measurement in processing parts.

### 5.2. Outlook

Machine learning and deep learning are accelerating the rapid development of intelligent applications in the industry [32,33,34]. Luo et al. [35] described a deep convolutional neural network (CNN)-based technique for the detection of micro defects on metal screw surfaces and the experiment results showed that the proposed technique can achieve a detection accuracy of 98%; the average detection time per picture was 1.2 s. Comparisons with traditional machine vision techniques, e.g., template matching-based techniques, demonstrate the superiority of the proposed deep CNN-based one. Huang et al. [36] proposed a compact CNN-based model and the experiments indicated CNNs can be compact and hardware-friendly for future applications in automated surface inspection (ASI). We believe that deep learning based on image perception will further optimize the robustness and adaptability of this study. In the future, we will conduct in-depth learning-based research on automatic tool setting and parts online detection to improve the intelligent manufacturing process of CNC machine tools.

## Figures and Tables

**Table 1:** Configuration of the experimental system.

No.	Name	Type	Quantity
1	Industrial camera	GS3-U3-91S6M-C	2
2	Industrial lens	V5028-MPY	2
3	No. 1 light source	SCS-RIU80-24B	1
4	No. 2 light source	SCS-BL20015-24W	1
5	Integrated machine	Equipped with Gigabit network port	1
6	CNC milling machine	PPCNC (1 μm)	1
7	Milling cutter	1 ± 0.0005 mm	1
8	Tool-setting workpiece	1 mm wire-electrode cutting T-groove	1

**Table 2:** Image coordinates and machine tool coordinate values when the cutting point moved along the *X*-axis.

CNC Machine Coordinate *XY* (mm)	Image Coordinate *uv* (pixel)
(24.800,8.000)	(1971.934, 1684.825)
(24.600,8.000)	(1707.255, 1684.813)
(24.400,8.000)	(1443.397, 1684.818)
(24.200,8.000)	(1179.629, 1684.806)
(24.000,8.000)	(915.859, 1684.798)
(23.800,8.000)	(625.089, 1684.812)

**Table 3:** Image and machine tool coordinates when the cutting point moved along the *Y*-axis.

CNC Machine Coordinate *XY* (mm)	Image Coordinate *uv* (pixel)
(25.000,8.000)	(2234.753, 1684.802)
(25.000,8.200)	(2234.746, 1421.133)
(25.000,8.400)	(2234.757, 1157.224)
(25.000,8.600)	(2234.758, 893.511)
(25.000,8.800)	(2234.738, 629.742)
(25.000,9.000)	(2234.744, 365.973)

**Table 4:** Image and machine tool coordinates when the feature point moved along the *Z* axis.

CNC Machine Coordinate *Z* (mm)	Image Coordinates *uv* (pix)
15.000	(787.256, 713.256)
15.200	(787.237, 736.363)
15.400	(787.238, 758.256)
15.600	(787.245, 780.867)
15.800	(787.252, 803.253)
16.000	(787.247, 826.653)

**Table 5:** The verification of the experimental data. (Data of No. 1 CCD, *k_x_* = 0.000758 mm/pix, *k_y_* = 0.000757 mm/pix).

*X*_0_, *Y*_0_ (mm)	*X*_1_, *Y*_1_ (mm)	*u*_0_, *v*_0_ (pix)	*u*’, *v*’ (pix)	*X_T_, Y_T_* (mm)	*x_v_, y_v_* (μm)
24.000, 8.000	24.156, 7.853	337.214, 1604.373	543.558, 1798.250	24.156, 7.853	0.459, 0.248
24.000, 8.000	24.201, 7.984	337.214, 1604.373	602.976, 1624.592	24.202, 7.985	0.513, 0.326
24.000, 8.000	24.237, 8.060	337.214, 1604.373	650.427, 1524.457	24.237, 8.060	0.492, 0.259
24.000, 8.000	24.246, 8.122	337.214, 1604.373	662.121, 1442.727	24.246, 8.122	0.356, 0.382
24.000, 8.000	24.311, 8.292	337.214, 1604.373	748.272, 1217.706	24.312, 8.293	0.682, 0.452
24.000, 8.000	24.330, 8.322	337.214, 1604.373	773.212, 1178.190	24.331, 8.323	0.593, 0.339
24.000, 8.000	24.358, 8.359	337.214, 1604.373	809.967, 1129.529	24.358, 8.359	0.462, 0.426
24.000, 8.000	24.382, 8.256	337.214, 1604.373	841.737, 908.861	24.383, 8.526	0.551, 0.458
*x_v_*	Mean: 0.513; Std: 0.097; RMS: 0.009		*y_v_*	Mean: 0.361; Std: 0.082; RMS: 0.007	

**Table 6:** The verification of the experimental data. (Data of No. 2 CCD, *k_z_* = 0.000782411 mm/pix)**.**

*Z*_0_ (mm)	*Z*_1_ (mm)	*u*_0_, *v*_0_ (pix)	*d’* (pix)	*Z_T_* (mm)	*z_v_* (μm)
15.000	15.542	787.256, 713.256	692.730	15.542	0.567
15.000	15.893	787.256, 713.256	1142.019	15.893	0.529
15.000	16.128	787.256, 713.256	1442.332	16.128	0.496
15.000	16.395	787.256, 713.256	1783.663	16.396	0.558
15.000	16.551	787.256, 713.256	1983.202	16.552	0.679
15.000	15.236	787.256, 713.256	302.296	15.236	0.520
15.000	14.536	787.256, 713.256	592.374	14.537	0.520
15.000	14.256	787.256, 713.256	950.425	14.256	0.377
*z_v_*	Mean: 0.361; Std: 0.082; RMS: 0.007				

**Table 7:** ConoPint-3-3Z79030 typical parameters table.

Objective lens type		25N	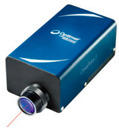
Standoff	mm	16	
Measurement range	mm	1	
Accuracy	μm	1	
Physical thickness range	mm	0.31–	
Linearity	±%	0.1	
X laser spot size	μm	5	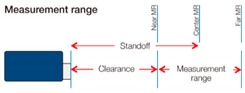
Measurement frequency	Hz	Up to 3000	
Weight	gr	700	
Power supply voltage		12 VDC±10%	

**Table 8:** Conopint-3 and vision system auxiliary measurement data table.

Vision System Measurement Value (μm)			ConoPint-3 Measurement Value (μm)			Comparison of Results V_T_ − V_T_’ (μm)		
*X_T_*	*Y_T_*	*d_T_*	*X_T_’*	*Y_T_’*	*d_T_’*	∆*X*	∆*Y*	∆*d*
240.952	724.925	181.996	241	725	182	−0.048	−0.075	−0.004
243.031	735.123	172.025	243	735	172	0.031	0.123	0.025
245.092	744.966	161.856	245	745	162	0.092	−0.034	−0.144
247.209	755.851	152.058	247	756	152	0.209	−0.149	0.058
249.132	764.868	142.210	249	765	142	0.132	−0.132	0.210
251.013	775.293	132.325	251	775	132	0.013	0.293	0.325
253.162	785.026	121.853	253	785	122	0.162	0.026	−0.147
255.251	795.258	112.726	255	795	111	0.251	0.258	−0.274
*x_v_*’: Mean: 0.117; Std: 0.086; RMS: 0.007			*y_v_*’: Mean: 0.136; Std: 0.097; RMS: 0.009			*z_v_*’: Mean: 0.148; Std: 0.117; RMS: 0.014		
